# RTA and LANA Competitively Regulate let-7a/RBPJ Signal to Control KSHV Replication

**DOI:** 10.3389/fmicb.2021.804215

**Published:** 2022-01-07

**Authors:** Chunhong Di, Guoxia Zheng, Yunheng Zhang, Enyu Tong, Yanli Ren, Yu Hong, Yang Song, Rong Chen, Xiaohua Tan, Lei Yang

**Affiliations:** ^1^Affiliated Hospital, Hangzhou Normal University, Hangzhou, China; ^2^School of Public Health, Hangzhou Normal University, Hangzhou, China

**Keywords:** Kaposi’s sarcoma-associated herpesvirus, replication and transcription activator, latency-associated nuclear antigen, let-7a, RBPJ

## Abstract

The recombination signal binding protein for immunoglobulin kappa J region (RBPJ) has a dual effect on Kaposi’s sarcoma-associated herpesvirus (KSHV) replication. RBPJ interaction with replication and transcription activator (RTA) is essential for lytic replication, while the interaction with latency-associated nuclear antigen (LANA) facilitates latent infection. Furthermore, our previous study found that LANA decreased RBPJ through upregulating miRNA let-7a. However, it is unclear whether RTA regulates the expression of RBPJ. Here, we show RTA increases RBPJ by decreasing let-7a. During KSHV replication, the RBPJ expression level was positively correlated with the RTA expression level and negatively correlated with the LANA expression level. The let-7a expression level was inverse to RBPJ. Knockdown of RBPJ inhibited the self-activation of RTA promoter and LANA promoter and weakened LANA’s inhibition of RTA promoter. Collectively, these findings indicate that RTA and LANA compete for let-7a/RBPJ signal to control the KSHV replication. Regulating the RBPJ expression level by RTA and LANA plays an important role during KSHV replication.

## Introduction

Kaposi’s sarcoma (KS) is the most common neoplasm of untreated acquired immunodeficiency syndrome (AIDS) patients. According to epidemiological and molecular data, Kaposi’s sarcoma-associated herpesvirus (KSHV) infection is the etiology of KS carcinogenesis. KSHV is also linked to two lymphoproliferative disorders, primary effusion lymphoma (Cesarman et al., 1995) and multicentric Castleman’s disease (Soulier et al., 1995). KSHV deploys two alternative genetic programs, latent and lytic in infection, during its infection of endothelial cells and B-lymphocytes (Chang et al., 1994). During latency, the circular KSHV episome is retained in the nucleus at a low copy number, and only a handful of viral genes are expressed, and no infectious progeny is produced. Most viral genes are expressed in an ordered cascade during lytic replication.

Both viral and host factors are involved in the regulation of KSHV infection status. Latency-associated nuclear antigen (LANA), encoded by the open reading frame 73 (ORF 73), is one of the few KSHV genes expressed in KSHV-infected cells during latency (Sun et al., 2014). LANA is a multifunctional protein that regulates transcription and cell growth (Lu et al., 2014; Wang et al., 2014; Gjyshi et al., 2015). LANA is the key viral protein in the maintenance of latent KSHV infection. The replication and transcription activator (RTA) encoded by ORF50 controls the switch from latent to lytic replication (Deng et al., 2007; Papugani et al., 2008; Zhao et al., 2015). RTA initiates the expression of the lytic genes in an ordered cascade and is both necessary and sufficient to trigger the lytic switch (Lukac et al., 1999; Gradoville et al., 2000). Coordination of RTA and LANA expression levels during the KSHV replication cycle is critical for effective latent and lytic replication of KSHV. However, the mechanism of coordination of these viral gene expression levels is still elusive.

Recombination signal binding protein for the immunoglobulin kappa J region (RBPJ) is a highly conserved, ubiquitously expressed protein and the major effector of Notch signaling (Wang et al., 2013). RBPJ has a dual effect on KSHV replication and is essential for KSHV lytic replication. The RBPJ deficient human B cells cannot produce progeny virions (Scholz et al., 2013). RBPJ binding to RTA is required to induce the expression of RTA itself, many lytic genes (Izumiya et al., 2009; Persson and Wilson, 2010; Wang et al., 2010a; Lu et al., 2011; Guito and Lukac, 2012). The binding of RTA to RBPJ also induces LANA transcription (Lan et al., 2005b). LANA, in turn, binds to RBPJ, which inhibits RTA expression, and this negative feedback contributes to the establishment and maintenance of the viral latency (Lan et al., 2005a,b, 2006; Lu et al., 2011). Thus, RBPJ functions as a hub of positive and negative feedback and a balancer to regulate KSHV lytic and latent replication.

miRNAs are short single-stranded RNA molecules, approximately 22 nucleotides in length. They are involved in the post-transcriptional regulation of gene expression by binding to complementary sequences in their target mRNAs’ 3’-UTRs. The let-7 miRNAs are tumor suppressor factors (Roush and Slack, 2008; Shimizu et al., 2010) and are also targeted by several viruses (Edge et al., 2008; Wang et al., 2010b; Cheng et al., 2013). Previously, we demonstrated that let-7a represses KSHV reactivation (Tan et al., 2015), and LANA upregulates let-7a and let-7a inhibits RBPJ by direct targeting its 3’UTR (Qi et al., 2019). As a result, the let-7a/RBPJ signal is defined as let-7a suppressing RBPJ expression. However, it is unclear whether RTA also regulates RBPJ expression. In the present study, we showed that RTA represses let-7a while upregulating RBPJ. RTA downregulates let-7a expression through upregulating NF-κB/Lin28B signaling pathway. During the KSHV replication cycle, the expression level of RBPJ positively correlated with that of RTA, while the expression level of Let-7a negatively correlated with that of RTA. Both RTA and LANA regulated the let-7a/RBPJ signal but in opposite directions. RBPJ is required for the cross-regulation of RTA and LANA, and knockdown RBPJ impaired RTA activation of RTA and LANA promoter and LANA inhibition of RTA promoter. Collectively, these results indicated that RTA and LANA compete to control the let-7a/RBPJ signaling pathway, which may contribute to coordinating the expression levels of these two key viral factors, playing an important role in maintaining latent infection and switching to lytic replication of KSHV. These results suggested that tightly controlling the expression of RBPJ is necessary for the KSHV life cycle.

## Materials and Methods

### Cell Lines and Plasmids

293T cells were cultured in Dulbecco modified Eagle medium (DMEM) medium supplemented with 10% (v/v) of FBS, 2 mM glutamine, 1 mM sodium pyruvate, 100 U/ml penicillin and 100 mg/ml streptomycin at 37°C under 5% CO_2_. Human microvascular endothelial cell 1 (HMEC-1) was cultured in MCDB-131 medium (MAC Gene Technology Co. Ltd., Beijing, China) supplemented with endothelial cell growth supplement (ECGS, Upstate Biotechnology, Lake Placid, NJ), and another condition is the same as 293T cells. The iSLK.RTA and iSLK.219 cells were maintained in DMEM containing G418 100 μg/ml, puromycin 4 μg/ml, hygromycin 100 μg/ml, another condition is the same as 293T cells.

The iSLK.RTA cells, iSLK cell line expresses a Dox-inducible RTA transgene. The iSLK.219 cells carry the recombinant KSHV clone called rKSHV.219, constitutively expressing GFP while encoding an RTA-inducible RFP reporter gene (Vieira and O’hearn, 2004). iSLK.219 cells express RTA under the control of a doxycycline (DOX)-responsive promoter.

The ORF73 (LANA) expression plasmid pCAGGS-LANA and the promoter-reporter plasmids of RTA (pRTA) and LANA (pLANA). The RTA expression plasmid pIRES2-EGFP was constructed as previously described (Tan et al., 2015). The plasmids were transfected into 293T, iSLK.219, or iSLK.RTA cells using lipofectamine (Invitrogen, Carlsbad, CA, United States). In this study, the pCAGGS-LANA and pIRES2-EGFP-ORF50 were also used as the standards for quantitative PCR (qPCR).

### rKSHV.219 Generation

For KSHV generation, iSLK.219 cells were cultured in a T150 flask till the confluent reached about 90%, doxycycline (DOX) was added to a final concentration of 500 ng/ml for 72 h, then, the cells and supernatant were collected separately by brief centrifugation. Collected cells were further lysed through freezing and thawing by adding liquid nitrogen 3 times. The pellet was re-suspended with the supernatant and filtered with a 0.22-μm filter to remove the debris and collect the flow. Then, it was subjected to centrifuge at 1,40,000 *g*, 4°C for 2 h, the supernatant was removed, and the pellet was subjected to air dry. The viruses were re-suspended in DMEM medium without antibiotics and FBS.

### Promoter Luciferase Reporter Assay

si-RBPJ or non-specific siRNA was transfected into 293T cells for 24 h, then pRTA or pLANA were co-transfected with pIRES2-EGFP-RTA, or pCAGGS-LANA, the mixture of pIRES2-EGFP-RTA and pCAGGS-LANA at different ratios to investigate the impact of RBPJ on RTA and LANA promoter activity. Cells were cultured in DMEM for 24 h, and then the cells were collected and analyzed for luciferase activity. Luciferase activity was measured using the dual-luciferase reporter assay kit (Promega, Madison, WI, United States) and normalized to Renilla luciferase activity and total protein level.

### RNA Inference Assay

The commercially synthesized siRNAs targeting RBPJ, RTA, si-p65, and Lin28B, or the non-specific control siRNAs as control (RiboBio, Guangzhou, China), were transfected into 293T cells or iSLK.RTA cells using riboFECT™ CP reagent (Ribo, Guangzhou, China). The knockdown efficiency was confirmed by detecting the targeted genes’ mRNA and protein levels.

### Quantitative Real-Time PCR

Total RNA from cells was reverse transcribed using the ThermoScript™ RT-PCR System (Invitrogen, Carlsbad, CA, United States). The mRNA quantity of LANA, RTA, and RBPJ was assayed by real-time PCR performed using the methods described previously (Takara Bio Inc. Otsu, Japan) with specific primers. Real-time PCR data were presented using the 2^-ΔΔCt^ method with β-actin or GAPDH as the internal control.

We also use the RTA and LANA plasmids as standards to quantify mRNA copies of RTA and LANA during lytic reactivation. We define the y value as the cycle of threshold (Ct) and log(molecules) of RTA or LANA. In this study, got the formula y = −3.448x + 34.37 (R = 0.9995) for RTA, and the formula y = −3.543 + 42.61 (R = 0.9991) for LANA. According to the formula, the mRNA copies of RTA and LANA were calculated.

Cells were harvested, and miRNAs were isolated using miRcute miRNA Isolation Kit (Tiangen Biotech, Beijing, China) to measure the production of let-7 miRNA. The let-7 miRNAs were quantified using the methods as described previously with commercial primers (RiboBio, Guangzhou, China). Data were analyzed by the 2^-ΔΔCt^ method with U6 as the internal control. The primer sequences for qPCR are listed in Table 1.

**Table 1:** Primer sequences for qRT-PCR.

Primer	Sequence (5'-3')
LIN28B-F	CATCTCCATGATAAACCGAGAGG
LIN28B-R	GTTACCCGTATTGACTCAAGGC
P65-F	CACAAGGCAGCAAATAGACG
P65-R	GAGTTAGCAGTGAGGCACCA
RBPJ-F	GACTCAGACAAGCGAAAGCA
RBPJ-R	TTTGGAAGGTTTGGAGATGAC
ORF73-F	GCCTACATCTCCCATCTCCA
ORF73-R	ATCCTCCTCGTCATCCTCCT
ORF50-F	CGCAATGCGTTACGTTGTTG
ORF50-R	GCCCGGACTGTTGAATCG
GAPDH-F	AATGGACAACTGGTCGTGGAC
GAPDH-R	CCCTCCAGGGGATCTGTTTG
Actin-F	GAGCGGGAAATCGTCCGTGACATT
Actin-R	GATGGAGTTGAAGGTAGTTTCGTG
let-7a-F	GCGCCTGAGGTAGTAGGTTG
let-7a-R	CAGTGCAGGGTCCGAGGT
U6-F	CTCGCTTCGGCAGCACATATACT
U6-R	ACGCTTCACGAATTTGCGTGTC

### Western Blot

All Western blots were probed with specific antibodies directed against β-Actin, RBPJ, LIN28B (Abcam, Cambridge, MA, United States), RTA (Abbiotec, Escondido, CA, United States), LANA (MBL Life Science, Japan), and p65 (CST Biotechnology, Santa Clara, CA, United States). β-Actin or GAPDH was used as the internal loading control.

### Statistical Analysis

All results are expressed as means ± standard errors of the means (SEMs) of experiments independently repeated at least three times. Unpaired Student’s *t* test was used to evaluate the statistical difference between samples. Significance was evaluated with *p* values and represented as follows: *, *p* < 0.05; **, *p* < 0.01; NS, nonsignificant.

## Results

### RTA Downregulates let-7a While Upregulating RBPJ

Our previous study showed that LANA increased let-7a through NF-κB/Lin28B signaling (Qi et al., 2019). LANA is the key factor in maintaining latent infection, while RTA is the switch of lytic replication. Therefore, we want to know whether the expression of let-7a and RBPJ also be regulated by RTA.

**Figure 1:**
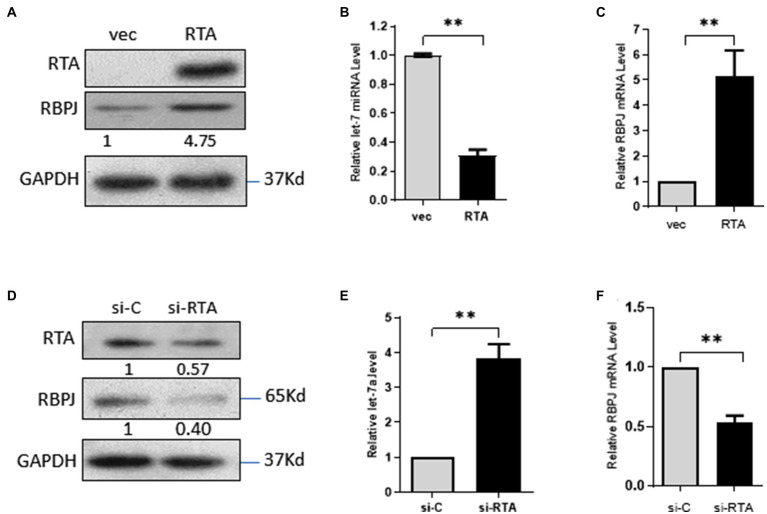
RTA decreases let-7a while upregulating RBPJ. **(A–C)** 293T cells were transfected with pIRES2-EGFP-RTA (RTA) or empty vector (vec) for 48 h. **(A)** Protein levels of RTA and RBPJ were determined by Western blot. GAPDH was the loading control. **(B)** miRNAs were extracted, and qPCR assays determined the let-7a levels with U6 as the internal control. **(C)** Total RNA was extracted, and qPCR assays detected RBPJ mRNA. GAPDH was the internal control. **(D-F)** iSLK.RTA cells were treated with doxycycline (Dox) for 36 h, si-RTA or non-specific (si-C) were transfected for 48 h. **(D)** Levels of RTA and RBPJ protein were determined by a Western blot assay. **(E)** miRNAs were extracted, and qPCR assays detected the let-7a levels with U6 as the internal control. **(F)** RBPJ mRNA levels were determined with a qPCR assay. The results shown are means ± SEM. *P* values were determined with Student’s *t* test. **p < 0.01.

293T cells were transfected with pIRES2-EGFP-RTA or the empty vector pIRES2-EGFP as control. The expression of RTA protein in the 293T cells was confirmed by Western blot (Figure 1A). Overexpression RTA decreases the expression of let-7a (Figure 1B). RBPJ is directly targeted by let-7a (Qi et al., 2019), so we also detected the RBPJ expression level. As we expected, RTA increased the expression of RBPJ at mRNA and protein levels (Figures 1A,C). To confirm the effect of RTA on the expression of let7a and RBPJ, iSLK.RTA cells, which express doxycycline (Dox) inducible RTA, were treated with Dox for 36 h to induce RTA expression, then siRNA specific targeting RTA or non-specific siRNA was transfected, the knockdown efficiency of RTA confirmed by Western blot (Figure 1D). Knockdown RTA in iSLK.RTA increased let-7a (Figure 1E) but decreased RBPJ′s mRNA and protein levels (Figures 1D,F).

### RTA Decreases let-7a Through NF-κB/Lin28B Signaling

**Figure 2:**
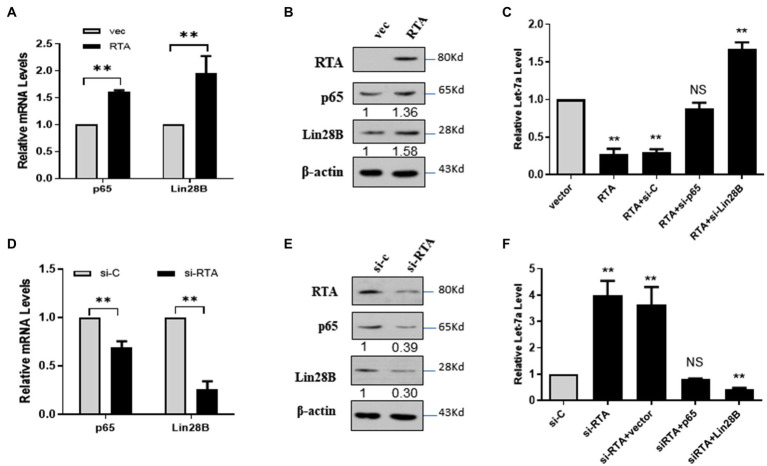
RTA decreases let-7a expression by upregulating NF-κB and LIN28B. **(A–C)** 293T cells were co-transfected with a plasmid expressing RTA and p65 or Lin28B. **(A)** p65 and Lin28B mRNAs were quantified with qPCR, and **(B)** The levels of RTA, p65, and Lin28B proteins were quantified with Western blot. **(C)** miRNAs were extracted, and the let-7a levels were determined with qPCR assays with U6 as the internal control. **(D–F)** iSLK.RTA cells were co-transfected with si-RTA and sip65 or si-Lin28B. **(D)** p65 and Lin28B mRNAs were quantified with qPCR, and **(E)** The RTA, p65, and Lin28B proteins were quantified with Western blot. **(F)** miRNAs were extracted, and qPCR assays determined the let-7a levels with U6 as the internal control. The results shown are means ± SEM. *P* values were determined by Student’s *t* test. **p < 0.01.

let-7a is a tumor suppressor miRNA whose expression is regulated by several signaling molecules, and the NF-κB/Lin28B signaling pathway is one of the most important pathways involved (Roos et al., 2015; Sangiao-Alvarellos et al., 2015; Joshi et al., 2016). Therefore, we investigated whether NF-κB/LIN28B signaling contributes to the RTA-induced downregulation of let-7a. Ectopic RTA in 293T cells increased the expression of P65 and Lin28B (Figures 2A,B). RTA decreased let-7a, and p65 knockdown rescued the expression of let-7a. The let-7a level in 293T cells co-transfected with RTA and si-lin28B was even higher than that of the control group (Figure 2C). These results indicate that RTA suppresses let-7a expression depending on p65 and Lin28B. To confirm the results, we detected p65 and Lin28B mRNA levels in iSLK.RTA cells after knockdown the RTA expression. Knockdown RTA decreases p65 and Lin28B expression levels (Figures 2D,E). Knockdown RTA increases let-7a, and this effect was canceled by co-transfect with p65 and even downregulated when co-transfect with Lin28B (Figure 2F). These results suggested that RTA decreased let-7a and increased RBPJ, partly dependent on NF-κB/Lin28B signaling pathway.

### The Expression Level of RBPJ Positively Correlated With That of RTA During *de novo* Infection

**Figure 3:**
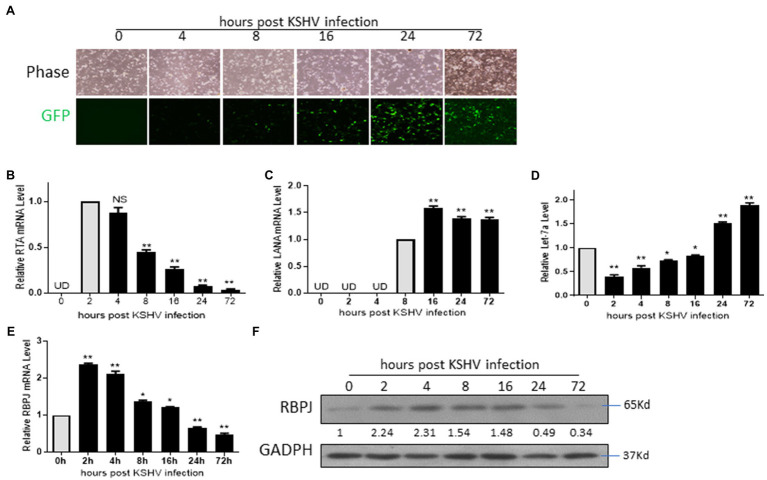
The expression level of RBPJ positively correlated with that of RTA during *de novo* infection. HMEC-1 cells *de novo* infected by rKSHV.219 and harvested at 0, 2, 4, 8, 16 24, and 72 h post-infection (hpi). **(A)** Cellular expression GFP was observed with inverted fluorescence microscopy. **(B)** The mRNA levels of RTA at indicated time points were determined by qPCR assays. **(C)** The mRNA levels of LANA at indicated time points were determined by qPCR assays. **(D)** The let-7a miRNA expression levels were determined by qPCR assays. **(E)** The RBPJ mRNA levels were determined by qPCR assays, and **(F)** protein and levels were determined by Western blot assay. UD: undetectable. The results shown are means ± SEM. *P* values were determined by Student’s *t* test. *p < 0.05, **p < 0.01.

Several studies have shown that KSHV *de novo* infection leads to a robust dynamic change in the cellular genes accompanying viral genes (Lan et al., 2005b; Gjyshi et al., 2015; Zou et al., 2017). Here, we want to investigate the expression time course of the viral and host cellular factors, including RTA, LANA and let-7a, RBPJ, in the *de novo* infected cells. The rKSHV.219 virions were generated from iSLK.219 cells. HMEC-1 cells were infected by rKSHV.219 and harvested at 0, 2, 4, 8, 16 24, and 72 h post-infection (hpi). GFP-positive cells could be observed at 4 hpi (Figure 3A). The mRNA of RTA could be detected as early as 2 hpi, and we defined the relative mRNA expression of RTA at this time point as “1.” After that, mRNA levels of RTA declined sharply. Compared with the mRNA level of 2 hpi, the mRNA level of RTA was 0.07 and 0.04 at 24 and 72 hpi, respectively. (Figure 3B). The mRNA expression of LANA could not be detected until 8 hpi (we defined the relative expression level of LANA at this time point as “1”). The mRNA expression level of LANA was even higher at 16 hpi and, after that, maintained at that level (Figure 3C). The let-7a expression levels decreased at 2 hpi and then increased gradually. During 2–16 hpi, the let-7a expression level is lower than the control, while at 24 and 72 hpi, its expression levels are higher than the control (Figure 3D). The RBPJ expression levels were increased at 2 hpi and then decreased gradually. During 2–16 hpi, the RBPJ expression level is higher than the control, while at 24 and 72 hpi, its expression levels are lower than the control (Figures 3E,F).

### The Expression Level of RBPJ Is Positively Correlated With That of RTA During Lytic Reactivation

**Figure 4:**
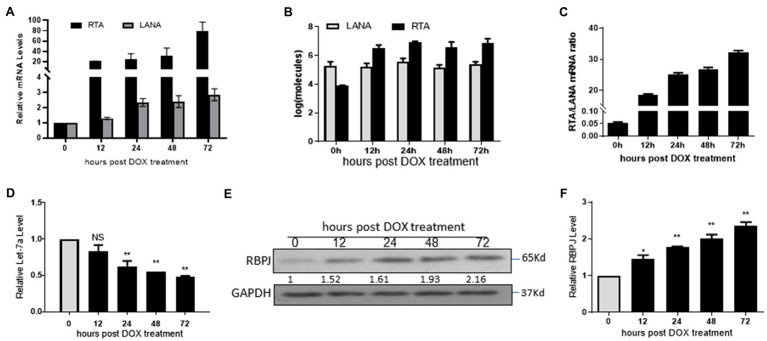
The expression level of RBPJ positively correlated with that of RTA during lytic reactivation. The iSLK.219 cells were treated with 500 ng/ml doxycycline (DOX) to induce lytic reactivation, and the cells were harvested at 12, 24, 48, and 72 h. **(A)** qPCR assays determined the mRNA levels of RTA and LANA. **(B)** The RTA and LANA mRNA copies were calculated according to the standard curve. **(C)** The RTA/LANA ratio was calculated according to their copies. **(D)** miRNAs were extracted, and qPCR assays determined the let-7a levels with U6 as the internal control. **(E,F)** qPCR assays and Western blot assay determined the relative mRNA and protein levels. The results shown are means ± SEM. *P* values were determined by Student’s *t* test. *p < 0.05, **p < 0.01.

The iSLK.219 cells were treated with 500 ng/ml doxycycline (DOX) to induce lytic reactivation, and cells were harvested at 12, 24, 48, and 72 h post DOX treatment. The let-7a expression level and the mRNA levels of RTA, LANA, and RBPJ were determined. During lytic reactivation, the expression of RTA increased dramatically, while the expression of LANA and RBPJ also increased slightly (Figure 4A). We also use the RTA and LANA plasmids as standards to quantify mRNA copies of RTA and LANA during lytic reactivation. We got the formula for calculating copy numbers based on the cycle of threshold (Ct) value. The mRNA copies of RTA and LANA were calculated according to the corresponding formula shown in the method section (Figure 4B). The RTA/LANA ratios were calculated based on RTA and LANA mRNA copies (Figure 4C). In the latently infected iSLK.219 cells, before DOX treatment, the expression of LANA is about 20 folds higher than RTA. However, after being induced by DOX, although both the expression of RTA and LANA increased, the RTA expression level increased more rapidly, the RTA/LANA ratio is 18.6 at 12 h post DOX treatment, and steadily increasing during lytic reactivation, at 72 h post dox treatment, the RTA/LANA ratio reach 32.2. During the lytic reactivation, the RTA/LANA ratio increased sharply, and the expression levels of let-7a decreased (Figure 4D), while RBPJ increased (Figures 4E,F).

### RTA and LANA Competitively Regulate let-7a/RBPJ Signal

**Figure 5:**
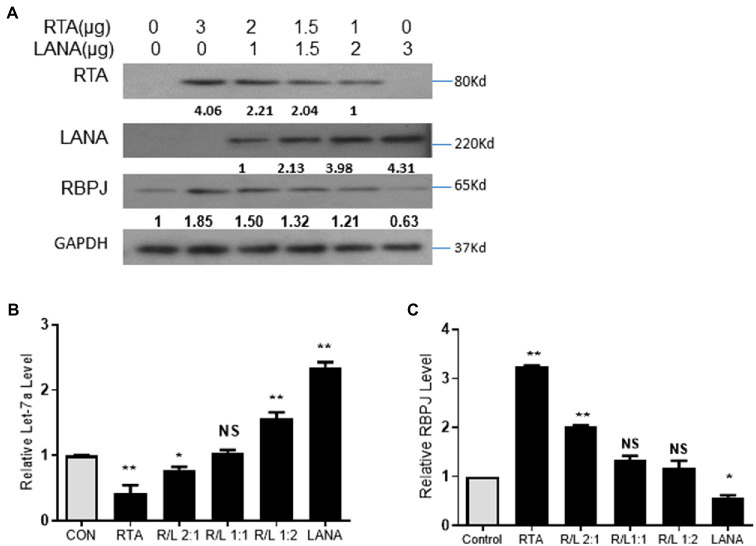
RTA and LANA competitively regulate let-7a/RBPJ signal. **(A)** Total 3 μg plasmids of RTA and LANA in indicated ratios were mixed and co-transfected into 293T cells. The protein levels of RTA, LANA, and RBPJ were determined by Western blot assay. **(B)** miRNAs were extracted, and qPCR assays determined the let-7a levels with U6 as the internal control. **(C)** Total RNA was extracted, and qPCR assays determined the levels of RBPJ mRNA. GAPDH was the internal control. The results shown are means ± SEM. *P* values were determined by Student’s *t* test. *p < 0.05, **p < 0.01.

During *de novo* infection and lytic reactivation, as the relative expression levels of RTA and LANA changed, the expression of let-7a and RBPJ changed accordingly. Here, we want to know whether the expression of let-7a and RBPJ is controlled by the RTA/LANA ratio. Total 3 μg plasmids of RTA and LANA in different ratios were mixed and co-transfected into 293T cells. The proteins level of RTA and LANA was determined by Western blot (Figure 5A). As the RTA/LANA ratio increased, the let-7a levels decreased (Figure 5B), while the mRNA and protein expression levels of RBPJ increased (Figures 5A,C).

### RBPJ Is a Necessary Factor for Cross-Regulation Between RTA and LANA

Both RTA and LANA bind to RBPJ and regulate the expression of RBPJ but in the inverse direction by regulating the expression of let-7a. RTA binds to RBPJ and upregulates LANA, while LANA binds to RBPJ and downregulates RTA. Here we show that RTA and LANA competitively regulate let-7a and RBPJ. We wondered whether RBPJ is an essential factor for this cross-regulation between RTA and LANA.

**Figure 6:**
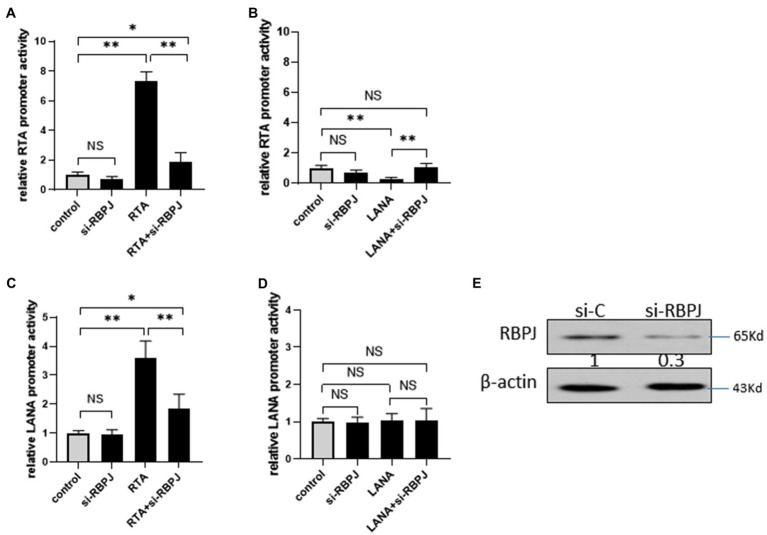
Knockdown RBPJ impaired the cross-regulation between RTA and LANA. **(A–B)** 293T cells were transfected with si-RBPJ or non-specific siRNA as control, 24 h later, these cells were co-transfected with RTA promoter-reporter plasmid pGL3-RTA, with RTA or LANA. At 24 h post-transfection, the cell lysate of each transfection was harvested for a luciferase assay. **(C–D)** 293T cells were transfected with si-RBPJ or non-specific siRNA as control. 24 h later, these cells were co-transfected with LANA promoter-reporter plasmid pGL3-RTA, with RTA or LANA. At 24 h post-transfection, the cell lysate of each transfection was harvested for a luciferase assay. **(E)** 293T cells were transfected with si-RBPJ or non-specific siRNA (si-C). 24 hour later, the protein levels of RBPJ and β-actin (as loading control) were determined by Western blot. The results shown are means ± SEM. *P* values were determined by Student’s t-test. *p < 0.05, **p < 0.01.

293T cells were transfected with si-RBPJ or non-specific siRNA as a control to investigate the impact of RBPJ on RTA promoter activity, and the knockdown efficiency was confirmed by Western blot (Figure 6E). 24 h later, these cells were co-transfected with RTA promoter-reporter plasmid pRTA, with RTA or LANA. Knockdown RBPJ does not affect RTA promoters’ activity. Ectopic expressed RTA induces the RTA promoter, but the RTA promoter activity is much lower than that in the RBPJ knockdown cells (Figure 6A). LANA represses the RTA promoter activity, and si-RBPJ canceled this effect (Figure 6B).

The effect of RBPJ on LANA promoters was also investigated. 293T cells were transfected with si-RBPJ or non-specific siRNA as control, 24 h later; these cells were co-transfected with LANA promoter-reporter plasmid pLANA, with RTA or LANA, or RTA and LANA expression plasmid mixture. RTA increases the LANA promoter activity, and knockdown of RBPJ reduces the activation amplitude induced by RTA but is still significantly higher than the control (Figure 6C). LANA does not affect LANA promoters’ activity with or without si-RBPJ (Figure 6D).

## Discussion

Viruses relying on the host cell for replication are obligate intracellular parasites. Virus replication involves different steps from a virus binds to the target cells until new progeny virions are made and released from the cells. For KSHV, the process of viral release from the cells results in the lysis of host cells. KSHV must carefully regulate both latent and lytic phases of its lifecycle to balance persisting in the infected cells and spreading to naïve cells. RTA and LANA are key viral factors that switch to lytic replication and maintain latent infection of KSHV, respectively. Coordination of RTA and LANA expression levels is critical for the KSHV replication cycle.

RTA is the switch of lytic reactivation, and LANA is an essential factor for latent infection. RBPJ acts as a balancer for the positive and negative feedback regulation, regulating the lytic and latent replication of KSHV. Previously, we demonstrated that LANA decreases RBPJ expression level through upregulating let-7a (Qi et al., 2019). Here, we show that RTA regulates the let-7a/RBPJ signal, but inversely, RTA downregulates let-7a and upregulates RBPJ. RTA suppresses let-7a expression while upregulating RBPJ. It is not a surprise that the expression level of RBPJ is tightly controlled during the KSHV life cycle.

The miRNAs are short single-stranded RNA molecules, approximately 22 nucleotides in length. RBPJ is directly targeted by miRNA let-7a (Qi et al., 2019). The present study shows that RTA induces p65 and Lin28B expression and may thus contribute to repressing let-7a. p65 induces the transcription of let-7a but does not increase the mature let-7a level because of induction of Lin28B (Wang et al., 2012). lin28B acts as a post-transcriptional repressor of let-7 biogenesis by binding to the loop portion of the let-7a precursor to inhibit the binding of Drosha or Dicer, thereby inhibiting their processing and reducing their processing the levels of mature let-7(Piskounova et al., 2011). LANA upregulates let-7a, but RTA downregulates let-7a. The expression level of Let-7a is a sensor of the expression level of RTA and LANA. By fine-tuning the expression of let-7a by RTA and LANA, the expression level of RBPJ was determined to maintain the balance between lytic and latent KSHV.

From the binding of KSHV to target cells to establish a latent infection state, the gene expression of both the virus and host changed dramatically. The immediately early gene, RTA, was expressed very soon after *de novo* infection, but its expression level decreased sharply. During the lytic reactivation, although the expression of RTA and LANA significantly increased, the expression levels of RTA increased more. Lan et al. (2005b) also reported that LANA was increased during lytic reactivation. The role of increased LANA in lytic replication remains unclear. LANA promotes cell survival and inhibits apoptosis (An et al., 2005; Curreli et al., 2005; Cai et al., 2006; Woodard et al., 2015), which may increase the lifespan of lytic replication cells, increasing the virion production.

The RTA/LANA ratio reversed during the lytic phase process, and let-7a and RBPJ expression levels changed accordingly. It indicates that the relative quantities of RTA and LANA determine the direction of the let-7a/RBPJ signal. During the replication cycle of KSHV, the expression level of RBPJ is positively correlated to the expression of RTA and negatively correlated to that of LANA.

Here the results of the present study show that RTA and LANA competitively regulate the expression of RBPJ. Knockdown RBPJ almost impaired the positive feedback that activates RTA promoter by RTA and attenuated negative feedback that inhibits RTA promoter by LANA. In KSHV latently infected iSLK.219 cells, the RTA/LANA reach 1/20. There are several mechanism may contribute block the RTA self-activation positive feedback. The expression of LANA is much higher than RTA, which may compete RBPJ or other transcript factors which needed for RTA to trigger the positive feedback. In latent status, RTA is covalently modified by poly (ADP-ribose; Chung et al., 2021) or physically interacting with sirtuin 1 (He and Gao, 2014; Li et al., 2014), which inhibits its transactivation activity.

**Figure 7:**
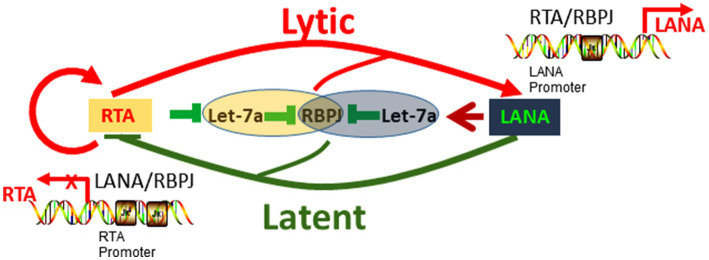
Hypothetical model of RTA and LANA competitively regulate let-7a/RBPJ signal to control KSHV replication.

In summary, the let-7a/RBPJ signal is competitively regulated by RTA and LANA, and the let-7a/RBPJ signaling direction is determined by the RTA/LANA ratio (relevant quantity of RTA and LANA). Through regulating let-7a, RTA increases RBPJ while LANA decreases RBPJ. RTA binds to RBPJ positive upregulates RTA, induces LANA transcription as well, in turn, LANA binds to RBPJ repress RTA promoter. There are two feedback loops, including the positive feedback loop that RTA binds RBPJ upregulates RTA and RBPJ, and the negative feedback loop that RTA upregulates LANA and LANA downregulates RTA. The coupled feedback loops interlocked at competitive regulation let-7a/RBPJ by RTA and LANA play an important role in the KSHV replication cycle (Figure 7).

## Data Availability Statement

The original contributions presented in the study are included in the article/supplementary material, further inquiries can be directed to the corresponding authors.

## Author Contributions

XT and LY designed the study. CD, GZ, YZ, and YR carried out the experiments. YZ and YR analyzed the data. CD drafted the manuscript, which all authors revised. All authors contributed to the article and approved the submitted version.

## Funding

This work was supported by the National Natural Science Foundation of China (81772168) and the Natural Science Foundation of Zhejiang Province (grant Nos. LQ18H190003 and LY12H16028).

## Conflict of Interest

The authors declare that the research was conducted without any commercial or financial relationships that could be construed as a potential conflict of interest.

## Publisher’s Note

All claims expressed in this article are solely those of the authors and do not necessarily represent those of their affiliated organizations, or those of the publisher, the editors and the reviewers. Any product that may be evaluated in this article, or claim that may be made by its manufacturer, is not guaranteed or endorsed by the publisher.
